# Polarization-Regularized Adversarial Pruning for Efficient Radio Frequency Fingerprint Identification on IoT Devices

**DOI:** 10.3390/s26062005

**Published:** 2026-03-23

**Authors:** Caidan Zhao, Haoliang Jiang, Jinhui Yu, Zepeng Meng, Xuhao He

**Affiliations:** Department of Informatics, Xiamen University, Xiamen 361102, China; jianghl@stu.xmu.edu.cn (H.J.); 23320211154261@stu.xmu.edu.cn (J.Y.); mengzp@stu.xmu.edu.cn (Z.M.); 23320241154618@stu.xmu.edu.cn (X.H.)

**Keywords:** radio frequency fingerprint, model compression, pruning, adversarial learning, polarization regularization

## Abstract

Radio frequency fingerprint identification (RFFI) based on physical-layer characteristics provides a reliable solution for secure authentication of Internet of Things (IoT) devices. Deep neural networks have demonstrated strong capability in improving RFFI performance; however, their high computational complexity and large parameter size pose significant challenges for deployment on resource-constrained edge devices. In RFFI tasks, existing pruning methods often lack effective performance recovery strategies, which leads to noticeable degradation in identification accuracy after pruning. To address this issue, this paper proposes a pruning method based on adversarial learning and polarization regularization. Polarization regularization is applied to learnable soft masks to effectively distinguish channels to be pruned from those to be retained. In addition, an adversarial learning-based performance recovery strategy is introduced to align the output feature distributions between the baseline network and the pruning network, thereby improving identification accuracy after pruning. Experimental results on multiple RFFI datasets demonstrate that the proposed method can effectively prune ResNet18 and VGG16, achieving substantial reductions in model complexity with only minor losses in identification performance.

## 1. Introduction

With the explosive growth of the number of Internet of Things (IoT) devices, the frequency of data interaction and the complexity of the network have increased significantly. As a result, IoT systems are facing tremendous information security threats and are vulnerable to malicious attacks such as spoofing and jamming, which can lead to information leakage [1]. Traditional IoT device security authentication is mainly implemented through key-based methods. However, key leakage problems are likely to occur during the stages of key generation, distribution, and rotation [2]. In comparison, radio frequency fingerprint identification (RFFI) technology based on the physical layer distinguishes identities according to the hardware characteristics of devices. These characteristics originate from hardware defects inherent in the manufacturing processes of electronic components. They possess features that cannot be counterfeited and remain stable over extended periods, independent of signal modulation schemes, transmitted information, or wireless channel characteristics [3]. Defects cause slight differences in signal transmission, which allows the identification of transmitting devices based solely on the transmitted signals. Therefore, RFFI technology can serve as a means of distinguishing the unique identity of devices, further resisting malicious network attacks, and enhancing the security of IoT systems [4].

Traditional RFFI methods primarily rely on manually designed physical-layer features. By extracting statistical quantities such as signal envelopes, spectrograms, and instantaneous amplitudes, combined with shallow classifiers, device identification is achieved [5]. However, this feature engineering approach depends on expert experience and is sensitive to channel conditions, noise, and device aging, resulting in insufficient generalization performance [6].

In recent years, deep learning has achieved breakthrough progress in fields such as speech, image, and natural language processing. RFFI methods based on deep learning can automatically extract robust data representations from signal data, significantly enhancing model identification accuracy and generalization capabilities [7,8]. However, deep learning models feature complex network structures, large parameter counts, and high computational resource demands [9], making direct deployment on edge embedded devices with limited processing and storage capabilities challenging. This contradiction becomes particularly pronounced in IoT scenarios characterized by dense nodes and energy constraints. Consequently, research on model compression is essential to reduce parameter counts, accelerate inference speeds, and enable efficient deployment of RFFI algorithms on edge devices [10,11].

In the field of text and image processing, model compression research primarily follows two technical approaches. The first involves designing inherently streamlined new architectures, such as MobileNet [12] and ShuffleNet [13], which employ separable or grouped convolution layers to achieve equivalent representations with reduced parameters and computational complexity. However, lightweight convolutional kernels are prone to underfitting when signal samples are scarce [14]. Moreover, redesigning and retraining specialized lightweight models not only requires lengthy cycles and complex debugging but may also compromise sensitivity to subtle hardware defects due to excessive simplicity [15]. Another approach involves post-training compression of existing models to eliminate redundant parameters and channels. Common model compression techniques include quantization [16], knowledge distillation [17], low-rank decomposition [18], and model pruning [19]. Quantization reduces model parameter precision—such as decreasing from 32-bit to 16-bit or 8-bit—to minimize the number of model parameters and memory consumption [20]. Direct application of quantization techniques causes numerical precision loss in identification model, making it unable to distinguish minor differences between different devices. This leads to a significant decline in the accuracy of RFFI [21,22]. The main idea of knowledge distillation is to transfer knowledge from the teacher model to the student model, making the student model approach the performance of the teacher model [23]. However, during the processing of radio frequency fingerprint features, the student model needs to retain sufficient radio frequency fingerprint details to maintain performance, resulting in a decline in the effect of model compression [24]. Low-rank decomposition decomposes the original complex large model into a lighter one by using matrices or tensors. However, this method requires complex decomposition operations and can only be applied layer by layer [25], which is not friendly to actual deployment. Model pruning refers to the process of removing redundant parameters from a model to obtain a sparse model, minimizing the computational load and parameter count of the model as much as possible [26].

Model pruning holds significant potential in compressing RFFI models. By directly removing redundant connections from a high-performance teacher network, it effectively preserves the core structure responsible for capturing and distinguishing subtle fingerprint features [21,27,28]. Jian et al. [27] pioneered the application of model pruning to RFFI model compression, employing progressive structural pruning to generate highly compressed neural networks with favorable compression rates across multiple identification models. Bothereau et al. [28] performed unstructured pruning on a convolutional neural network (CNN) across four RFFI datasets, achieving effective model compression. Zhu et al. [21] introduced pruning into the LoRa identification model compression workflow, successfully reducing model parameters by over 70%. However, these approaches lack mechanisms for restoring model performance post-pruning, resulting in significant degradation of identification accuracy under noisy conditions. Facing the challenges of reduced resolution in radio frequency fingerprint features under complex electromagnetic environments and increased demands for model robustness and generalization capabilities in identification tasks, there is an urgent need to achieve synergistic optimization between model compression and identification accuracy.

In response to this challenge, we propose a pruning-based compression algorithm for RFFI models, termed Adversarial Learning and Polarization Regularization based Pruning (AL-PP). The main contributions of this work are as follows:

1. To address the challenge of directly deploying deep neural network models on edge devices, we establish a framework for neural network model pruning. A soft mask layer is added to the target model. The distribution of soft mask values is encouraged to become sparse through polarization regularization, and channels with lower soft mask values are pruned to achieve model compression.
2. To mitigate the degradation in identification accuracy caused by model pruning, we introduce an adversarial learning-based performance recovery strategy. By aligning output features, we further enhance the identification accuracy of pruned models. The proposed algorithm is validated under signal-to-noise ratio (SNR) conditions ranging from −15 dB to 20 dB, and the pruned model maintains favorable performance.
3. We evaluate the proposed algorithm on multiple radio frequency fingerprint datasets including the LoRa dataset, IoT dataset, and ADS-B dataset. The pruned model was validated on the Jetson TX2 embedded device, demonstrating excellent performance.

## 2. Related Work

### 2.1. Model Pruning Techniques

Model pruning techniques can be categorized into unstructured pruning [29] and structured pruning [30]. Unstructured pruning can significantly reduce the complexity of typical convolutional neural network models [31]. However, unstructured pruning sparsifies weight parameters, generating numerous zero values in the weight matrix. This makes efficient utilization by mainstream hardware challenging and requires specialized sparse computation libraries, resulting in relatively limited application scenarios [32]. Structured pruning prunes entire structural units, preserving the pruned model’s regular network topology. This approach is more hardware-friendly and suitable for resource-constrained edge devices [33]. This property is particularly important for RFFI applications, where models often need to be deployed under limited computational resources while still preserving subtle device-specific fingerprint features.

Structured pruning algorithms aim to reduce model complexity by pruning less significant channels within the network, thereby achieving model compression. Consequently, selecting an appropriate metric to assess channel importance becomes particularly critical. Currently, common approaches primarily utilize the magnitude of the channel norm to directly reflect its importance [34], or introduce additional parameters for channels that evolve during training, ultimately evaluating channel importance based on parameter values [35]. Among these, a widely adopted method correlates channels with the scaling factor γ of the Batch Normalization (BN) layer, using the absolute value of γ to determine channel importance [36]. Specifically, $\ell_{1}$ regularization is applied to γ, as shown in the following equation:

(1) $$R\left(\gamma \right)=\lambda {∥\gamma ∥}_{1}$$

where R denotes the regularization term, λ represents the sparsity factor, and ${∥\gamma ∥}_{1}$ indicates the application of $\ell_{1}$ regularization to the scaling factor. During training, the distribution of γ factors gradually becomes sparser. The magnitude of the γ factor reflects the importance of the corresponding channel. Upon training completion, channels associated with smaller γ values can be pruned based on the pruning threshold.

However, $\ell_{1}$ regularization tends to push all scaling factors toward zero, making it difficult to distinguish between channels that should be pruned and those that should be retained, thereby complicating the identification of an appropriate pruning threshold. This limitation is more critical in RFFI, where useful discriminative information is embedded in subtle device-specific signal characteristics [4,8]. If informative channels are not clearly separated from redundant ones during pruning, important RF fingerprint features may be weakened or removed. Therefore, a more appropriate regularization method should be selected to separate the channels to be pruned from those to be retained.

Different from $\ell_{1}$ regularization, polarization regularization [37] polarizes the scaling factor into zero and positive values to form a clear boundary, suppressing only some channels while retaining the rest intact. Given *n* layers of scaling factors $\gamma =(\gamma_{1},\gamma_{2},\ldots ,\gamma_{n})$, polarization regularization is defined as:

(2) $$R\left(\gamma \right)=\mu {∥\gamma ∥}_{1}-{∥\gamma -\bar{\gamma }∥}_{1}$$

where γ denotes the set of Batch Normalization (BN) scaling factors across all channels, $\bar{\gamma }$ denotes the global mean of all scaling factors computed over all channels, and μ denotes the polarization parameter that controls the strength of the polarization regularization. It should be noted that the polarization regularization in Equation (2) does not simply drive all scaling factors toward zero. The first term introduces an overall sparsity tendency through an $\ell_{1}$ regularization, while the second term explicitly maximizes the deviation of each scaling factor from the global mean. In practice, channels with scaling factors close to zero indicate negligible contribution and can be safely pruned during structured pruning.

Under the joint effect of these two terms, the scaling factors are encouraged to exhibit a bimodal distribution: unimportant channels are driven toward values close to zero, whereas important channels are pushed away from the mean and retain relatively large values. This polarization behavior enables a clear separation between channels to be pruned and those to be retained.

Existing structured pruning methods mainly focus on identifying redundant channels or filters and improving compression efficiency through importance estimation and structured removal [32,38,39]. Most of these methods are developed and evaluated in general vision tasks. Although some studies have recognized that coarse-grained structured pruning may incorrectly remove still-useful units [38], their optimization is still centered on compression effectiveness and computational efficiency rather than the preservation of task-specific discriminative representations. Recent studies have also begun to investigate pruning for RFFI models [21,27,28]. In this task, model compression needs to consider not only redundant channel removal, but also the preservation of informative output representations related to device-specific fingerprint discrimination. From this perspective, existing pruning pipelines provide useful foundations, but the feature-preservation requirement of RFFI under model compression has not yet been fully studied.

### 2.2. Adversarial Learning

Generative Adversarial Networks (GANs) [40] serve as effective deep generative models capable of learning complex high-dimensional data distributions without relying on prior assumptions. A typical GAN consists of two components: a generator and a discriminator. The generator produces new data that closely resembles real target data; the closer the generated data distribution aligns with the real data distribution, the better the generator’s performance. The discriminator distinguishes between the generated new data and the real data. Formally, let *G* denote the generator, *D* denote the discriminator, *z* denote a random noise vector sampled from a prior distribution $P_{z}\left(z\right)$, and *x* denote real data samples drawn from the data distribution $P_{\mathrm{data}}\left(x\right)$. The objective function of a conventional GAN is defined as:

(3) $$\min_{G}\max_{D}\;F\left(D,G\right)=E_{x∼P_{\mathrm{data}}}\left[\log D(x)\right]+E_{z∼P_{z}}\left[\log\left(1-D(G(z))\right)\right]$$

During training, the discriminator is optimized to maximize its ability to correctly classify real and generated samples, while the generator is optimized to produce samples that are indistinguishable from real data, thereby minimizing the above objective.

In this work, adversarial learning is adopted as a mechanism for global output distribution alignment during pruning. Since channel removal may weaken the representation capacity of the compressed network, the resulting output distribution can deviate from that of the baseline model. This issue is particularly important for RFFI, where reliable identification depends on sufficiently rich and discriminative feature representations. By encouraging the pruned network to match the global output distribution of the baseline model, adversarial learning helps compensate for the loss of useful features caused by pruning and supports performance recovery. Specifically, the generator corresponds to the pruning network $f_{g}\left(\cdot ;M_{G},m\right)$, while the discriminator corresponds to $D(\cdot ;M_{D})$. Different from conventional GANs that generate samples from random noise, the “fake” samples in our framework are the outputs of the pruning network, whereas the “real” samples are the outputs of the fixed pre-trained baseline network $f_{b}\left(\cdot ;M_{B}\right)$.

### 2.3. Output Feature Alignment

The outputs of a neural network contain useful decision-related information for each input sample. Since the baseline network is not affected by model sparsification, its outputs preserve more complete information than those of the pruned model and can therefore be used to guide the pruning network during training. This guidance is useful for improving the consistency between the two networks and enhancing the identification performance of the pruned model.

Mean Squared Error (MSE) is a commonly used loss function in regression problems, measuring the average squared difference between actual and predicted values. Its calculation formula is as follows:

(4) $$\mathrm{MSE}=\frac{1}{n}\sum_{i=1}^{n}{∥x_{i}-y_{i}∥}^{2}$$

where *n* denotes the number of samples, and $x_{i}$ and $y_{i}$ denote the corresponding outputs to be aligned. In this work, the MSE is computed between the outputs of the baseline network and those of the pruning network under the same input sample. Unlike adversarial learning, which constrains the outputs at the distribution level, MSE provides sample-level supervision and helps the pruned model better preserve the local output structure learned by the baseline model.

## 3. Proposed Method

Unlike general model pruning methods that mainly pursue model compression, the proposed AL-PP framework is designed for RFFI, where discriminative cues are subtle, device-specific, and highly sensitive to channel removal. Therefore, the proposed method not only learns structured sparsity for model compression, but also incorporates feature-preserving mechanisms to maintain RF fingerprint discriminability during pruning.

**Figure 1:**
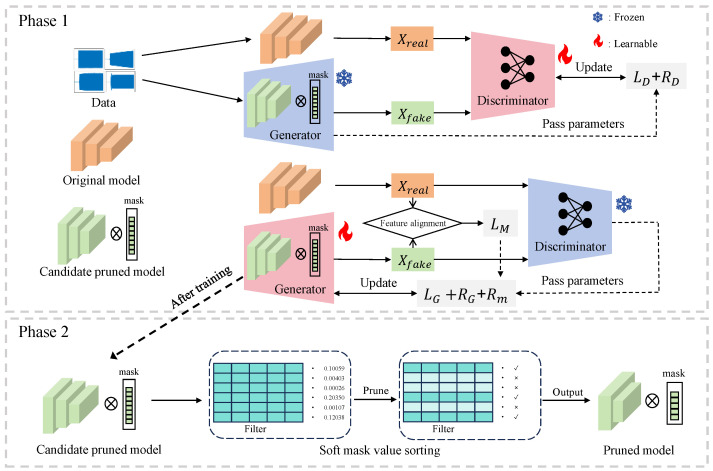
Overall framework of AL-PP. In the figure, blue and red modules represent the frozen and learnable states, respectively. Solid arrows indicate the computational flow, while thin dashed lines denote logical connections, and the thick dashed arrow signifies the phase transition.

Figure 1 illustrates the identification flowchart of the proposed adversarial learning-based polarized pruning algorithm. The original pre-trained model maintains fixed parameters throughout training. The pruning model initializes parameters identically to the original model, with an additional soft masking layer added to measure the importance of each neural network channel. During training, polarization regularization is applied to the mask, gradually inducing sparsity and pruning irrelevant channels. During neural network sparsification, model performance will experience a noticeable decline. To minimize performance loss and bring the pruning model’s identification accuracy close to the original model, a pruning performance recovery strategy combining adversarial learning and output feature alignment is adopted. Through adversarial learning training, the pruned network serves as the generator. During the adversarial game, it continuously approximates the original model’s output, enhancing the identification accuracy of the pruned model. By aligning the outputs of the original and pruned networks using MSE loss, the probability distributions of the pruned network’s outputs are made as similar as possible to those of the original network. After training, a pruned model is obtained with limited identification accuracy loss, significantly lower computational complexity, and fewer parameters than the original model.

### 3.1. Pruning Strategy

In RFFI tasks, discriminative information is often encoded in subtle device-specific channel responses rather than in dominant semantic features as in conventional vision tasks. Direct channel pruning may therefore remove channels that carry fine-grained RF fingerprint characteristics. To better distinguish informative channels from redundant ones, we introduce a soft-mask-based structured pruning mechanism and employ polarization regularization to drive mask values toward a more separable distribution.

To realize this objective, unlike the approach of directly applying polarization regularization to the scaling factor γ [37], we apply polarization regularization to the soft mask *m*. The soft mask is a continuous variable with values in the range [0, 1], which can be optimized jointly with other model parameters through backpropagation. During training, the soft mask values dynamically adjust as the loss function optimizes, so that channels contributing more to RF fingerprint representation tend to obtain larger mask values, whereas less informative channels tend to be suppressed. The polarization regularization method is expressed as follows:

(5) $$R_{m}\left(m\right)=\lambda \left(\mu {∥m∥}_{1}-{∥m-\bar{m}∥}_{1}\right)$$

where $R_{m}\left(m\right)$ represents the soft mask regularization term, λ denotes the sparsity factor, and μ is the polarization parameter. The $\ell_{1}$ norm ${∥m∥}_{1}$ introduces sparsity on the soft mask vector *m*, and $\bar{m}$ denotes the global mean of all soft mask values computed over all channels.

The regularization term consists of two components: one introduces an overall sparsity tendency through $\ell_{1}$ regularization, while the other explicitly maximizes the deviation of each mask value from the global mean $\bar{m}$, thereby amplifying the importance disparity among channels. From the perspective of hyperparameter design, the sparsity factor λ controls the overall strength of the pruning constraint, while the polarization parameter μ adjusts the tendency of the soft-mask values to separate into two distinct groups. If λ is too small, the regularization effect may be insufficient to suppress redundant channels; if it is too large, channels that still contribute to RF fingerprint representation may be excessively penalized. Similarly, if μ is too small, the mask values may remain concentrated around the global mean and fail to form a clear separation, whereas an excessively large μ may lead to overly aggressive polarization and weaken the preservation of subtle device-specific features. Therefore, these hyperparameters are selected to balance structured sparsity and feature preservation in RFFI tasks.

Under the joint effect of these two terms, the soft mask parameters gradually exhibit a clear bimodal distribution, where redundant channels are associated with mask values close to zero, and critical channels are assigned mask values close to one. This property provides a stable basis for channel-level structured pruning and helps preserve channels that are more relevant to RF fingerprint representation during model compression.

Compared with BN scaling factors, the soft mask provides a more direct and explicit measure of channel importance. Its dynamic learning behavior, combined with polarization regularization, enables more reliable channel selection for RFFI models, thereby achieving a better trade-off between model compression and the preservation of device-specific identification characteristics.

### 3.2. Model Performance Recovery Strategy

During training, polarization-regularized pruning progressively suppresses some channels, which may distort the output representation of the network and weaken subtle device-specific RF fingerprint cues. In RFFI tasks, such degradation is particularly harmful because identification depends on preserving fine-grained inter-device differences rather than only maintaining coarse prediction accuracy. To alleviate this problem, we introduce an adversarial-learning-based recovery strategy together with output feature alignment, so that the pruned network can remain consistent with the baseline model at both the distribution level and the sample level.

Specifically, adversarial learning [40,41] is used to align the overall output feature distribution of the pruned network with that of the unpruned baseline network. In this framework, the output of the original network is regarded as a real sample, the pruning network is regarded as the generator, and a shallow neural network is introduced as the discriminator. Through adversarial training, the pruning network is optimized to reduce the discrepancy between its outputs and those of the baseline model, thereby promoting performance recovery after pruning.

Let $f_{b}\left(\cdot ;M_{B}\right)$ denote the fixed pre-trained baseline network with parameters $M_{B}$, and let $f_{g}\left(\cdot ;M_{G},m\right)$ denote the pruning (generator) network with parameters $M_{G}$ and learnable soft masks *m*. For an input sample *x*, the corresponding output representations of the two networks are denoted by $y_{b}=f_{b}\left(x;M_{B}\right)$ and $y_{g}=f_{g}\left(x;M_{G},m\right)$. The discriminator is denoted by $D(\cdot ;M_{D})$, where $M_{D}$ represents its parameters. In addition, R(·) denotes regularization terms, where the subscript specifies the corresponding target variable. Unless otherwise specified, all expectations are taken over the training samples *x*, and *n* denotes the number of samples used in the MSE loss.

Based on this adversarial formulation, the discriminator is trained to distinguish the outputs of the baseline and pruned networks, while the pruning network is optimized to approximate the output characteristics of the baseline model. The discriminator loss function is expressed as follows:

(6) $$L_{D}\left(M_{D}\right)=E_{x}\left[\log D\;\left(f_{b}\left(x;M_{B}\right);M_{D}\right)\right]+E_{x}\left[\log\left(1-D\;\left(f_{g}\left(x;M_{G},m\right);M_{D}\right)\right)\right]$$

The discriminator loss function measures the ability of the discriminator to distinguish between the outputs of the original network and the pruning network. By maximizing $L_{D}\left(M_{D}\right)$, the discriminator is trained to assign high confidence to the outputs of the original network and low confidence to those of the pruning network. The generator loss function is expressed as follows:

(7) $$L_{G}\left(M_{G},m\right)=E_{x}\left[\log\left(1-D\;\left(f_{g}\left(x;M_{G},m\right);M_{D}\right)\right)\right]$$

The generator loss encourages the pruning network to fool the discriminator. By minimizing $L_{G}\left(M_{G},m\right)$, the pruning network updates its parameters $M_{G}$ and soft masks *m* so that its output distribution approaches that of the original network.

Additionally, we apply $\ell_{2}$ regularization to the weights of the pruned network to constrain parameter magnitude during pruning. This regularization helps stabilize optimization, reduces the risk of overfitting, and supports the preservation of reliable feature representations in the compressed RFFI model. The regularization term is defined as follows:

(8) $$R_{G}\left(M_{G}\right)=\frac{1}{2}{∥M_{G}∥}_{2}^{2}$$

In this work, adversarial learning serves as a distribution-level feature alignment mechanism to mitigate the representation degradation caused by structured pruning. By introducing a discriminator to distinguish the outputs of the baseline network from those of the pruned network, the pruning network is encouraged to gradually match the overall output feature distribution of the baseline model through adversarial training. This global alignment helps preserve the structural characteristics of RF fingerprint representations and reduces the loss of discriminative information caused by channel removal. As a result, adversarial learning provides effective supervision for recovering the representation capability of the pruned RFFI model.

To prevent the discriminator from dominating the training process, an adversarial regularization is also imposed on it, as shown in the following equation:

(9) $$R_{D}\left(M_{D}\right)=E_{x}\left[\log D\;\left(f_{g}\left(x;M_{G},m\right);M_{D}\right)\right]$$

Although adversarial learning aligns the output distributions at a global level, it does not explicitly constrain the consistency between the two networks for the same input sample. We use the MSE loss to align their outputs at the sample level. This constraint is important for RFFI, because preserving subtle output differences related to device-specific fingerprints helps maintain inter-device discriminability after pruning. The loss function is defined as follows:

(10) $$L_{M}\left(M_{G},m\right)=\frac{1}{2n}\sum_{x}{∥f_{b}\left(x;M_{B}\right)-f_{g}\left(x;M_{G},m\right)∥}^{2}$$

By minimizing $L_{M}\left(M_{G},m\right)$, the pruning network is encouraged to preserve the discriminative characteristics of the original network, especially the subtle output structures associated with device-specific RF fingerprints, which helps improve identification accuracy after pruning.

In the overall objective, the adversarial term is used to align the global output distribution of the pruned network with that of the baseline model, while the MSE term enforces sample-level output consistency. At the same time, the mask regularization term promotes structured sparsity, and the $\ell_{2}$ regularization term helps stabilize the training of the pruned network. During training, the discriminator parameters $M_{D}$ are updated by maximizing $L_{D}\left(M_{D}\right)+R_{D}\left(M_{D}\right)$, while the pruning network parameters $M_{G}$ and *m* are updated by minimizing the following objective:

(11) $$L_{G}\left(M_{G},m\right)+L_{M}\left(M_{G},m\right)+R_{m}\left(m\right)+R_{G}\left(M_{G}\right).$$

### 3.3. Algorithm Flow

The overall AL-PP procedure is illustrated in Algorithm 1. The proposed method is organized into two stages to preserve RF fingerprint discriminability during model compression: feature-preserving sparsification and structured channel pruning. In the first stage, the training data are fed into both the baseline model and the pruning candidate model equipped with soft masks to obtain their corresponding outputs. These outputs are then used to optimize the discriminator and the pruning network in an alternating manner. Through iterative adversarial training together with output feature alignment, the pruning candidate model gradually learns structured sparsity while maintaining consistency with the baseline model in terms of RF-related output characteristics.

During the sparsification stage, the soft-mask values in the pruning candidate model become increasingly polarized under the effect of polarization regularization, and the corresponding low-importance channels are gradually suppressed. Meanwhile, under adversarial supervision and output feature alignment, the pruned network is encouraged to preserve the output characteristics of the baseline model. As a result, the learned mask values more reliably reflect which channels contribute little to RF fingerprint representation, allowing redundant channels to be distinguished from those that are important for device-specific identification.

In the second stage, the learned soft-mask values obtained from the feature-preserving sparsification process are sorted to evaluate channel importance. According to the specified pruning ratio, channels with small mask values are removed, while channels with large mask values are retained. In this way, the final compact network is obtained by pruning channels that are less relevant to RF fingerprint identification.
**Algorithm 1** Flow of the proposed AL-PP algorithm**Input:** Training dataset $X=\{x_{1},x_{2},\ldots ,x_{n}\}$, where *n* denotes the number of training samples; fixed pre-trained baseline network $f_{b}\left(\cdot ;M_{B}\right)$; pruning network $f_{g}\left(\cdot ;M_{G},m\right)$; discriminator $D(\cdot ;M_{D})$; sparsity factor λ; polarization parameter μ; maximum number of training epochs *N*.1:**Phase 1: Sparsity training with performance recovery**2:Load the parameters of the pre-trained baseline network $M_{B}$.3:Initialize the pruning network parameters $M_{G}$ and the soft masks *m*, as well as the discriminator parameters $M_{D}$.4:**for** epoch=1 to *N* **do**5:   Fix baseline network $f_{b}\left(\cdot ;M_{B}\right)$ and update discriminator $D(\cdot ;M_{D})$6:   Feed the training samples x∈X into the baseline network and the pruning network to obtain the outputs $y_{b}=f_{b}\left(x;M_{B}\right)$ and $y_{g}=f_{g}\left(x;M_{G},m\right)$.7:   Update the discriminator parameters $M_{D}$ by maximizing the following objective using stochastic gradient descent (SGD):$$\max_{M_{D}}\;L_{D}\left(M_{D}\right)+R_{D}\left(M_{D}\right)$$8:   Fix discriminator $D(\cdot ;M_{D})$ and update pruning network $f_{g}\left(\cdot ;M_{G},m\right)$9:   Update the pruning network parameters $M_{G}$ and the soft masks *m* by minimizing the following objective using SGD:$$\min_{M_{G},m}\;L_{G}\left(M_{G},m\right)+L_{M}\left(M_{G},m\right)+R_{m}\left(m\right)+R_{G}\left(M_{G}\right)$$10:**end for**11:**Phase 2: Structured channel pruning**12:Evaluate the importance of each channel according to the learned soft mask values.13:Retain channels with large mask values and prune channels with small mask values to obtain the compact pruned model.**Output:** The compact model after structured pruning.

## 4. Results and Discussion

To validate the effectiveness of the proposed algorithm, experiments were conducted on a LoRa dataset, an IoT dataset, and a publicly available Automatic Dependent Surveillance–Broadcast (ADS-B) dataset [42]. To further demonstrate the generalization capability of the proposed algorithm, additional experiments were performed on the Canadian Institute For Advanced Research 10 (CIFAR-10) dataset [43]. In addition, hyperparameter studies were carried out to evaluate the effects of the sparsity factor λ and the polarization parameter μ on the identification accuracy and computational complexity of the pruned network, thereby further verifying the robustness and adaptability of the proposed method. To assess the performance of the pruned model in practical applications, its performance was also evaluated under noisy environments. Finally, the pruned model was deployed on an embedded device to validate its actual operational performance in resource-constrained environments.

### 4.1. Radio Frequency Fingerprint Dataset

**Figure 2:**
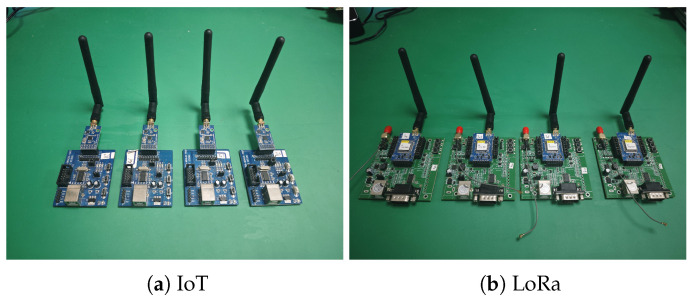
Two wireless transmitters.

We employed the F8L10A LoRa wireless communication module (Xiamen Four-Faith Communication Technology Company Limited, Xiamen, China) and the E05-MLE124AP2 IoT module (Chengdu Ebyte Electronic Technology Company Limited, Chengdu, China), which are widely used in IoT communication scenarios, as the transmitting devices. The E05-MLE124AP2 is a plug-in 2.4 GHz wireless module. The two wireless transmitters are shown in Figure 2. The blue transmitter board on the left corresponds to the E05-MLE124AP2 IoT module, while the green transmitter board on the right corresponds to the LoRa module.

**Figure 3:**
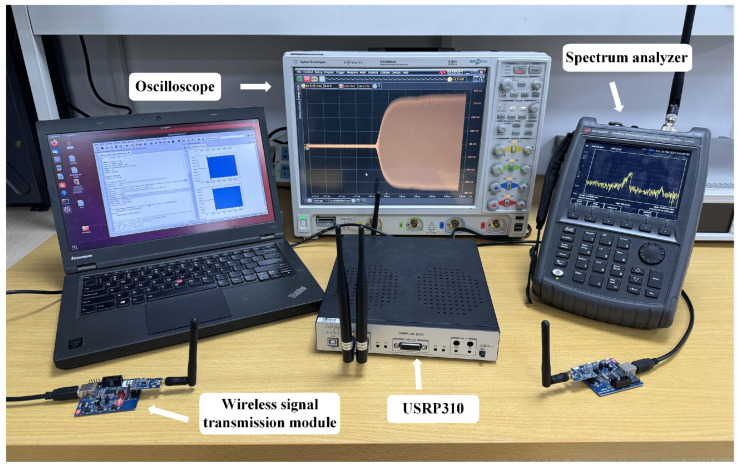
Data collection setup.

A Universal Software Radio Peripheral X310 (USRP X310, Wuhan Luoguang Electronics Company Limited, Wuhan, China), operated under the USRP Hardware Driver version 3.14.1, was used for signal acquisition to ensure flexibility and scalability. A wireless RF signal acquisition system was implemented on the GNU Radio platform (version 3.7.13.5), and the experimental setup is shown in Figure 3. A USRP X310 was employed to collect signals from 10 LoRa communication modules, with the center frequency set to 433 MHz. The sampling rate, sampling bandwidth, and antenna gain were 10 MHz, 1 MHz, and 10 dBi, respectively. The USRP X310 was also used to collect signals from 18 IoT devices, with the center frequency set to 2.4 GHz, and the sampling rate and sampling bandwidth were 40 MHz and 10 MHz, respectively.

**Figure 4:**
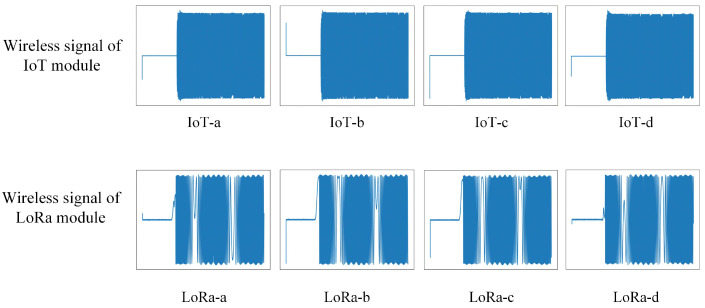
Time-domain waveforms of preprocessed signals.

Before RF fingerprint feature extraction, the acquired signals undergo several preprocessing steps, including start-point detection, signal segmentation, and data normalization. After completing all preprocessing procedures, a complete dataset is obtained. Figure 4 illustrates the time-domain waveforms of the preprocessed signals.

Automatic Dependent Surveillance–Broadcast (ADS-B) is a surveillance technology in which airborne equipment broadcasts aircraft four-dimensional position information along with identification data to enable air traffic monitoring and control [42]. The dataset was recorded using a USRP B210 at 1090 MHz with a sampling rate of 8 MHz over a 24-h period, collecting signals from more than 130 aircraft. Due to insufficient samples in some categories, 28 classes were selected for identification to ensure reliable network training performance.

### 4.2. Performance Evaluation of Proposed Algorithm

To effectively evaluate the performance of the proposed pruning algorithm, the widely used ResNet [44] and VGG16 [45] neural networks were adopted as baseline models for compression. On the RF fingerprint dataset, the models were trained for 150 epochs using stochastic gradient descent (SGD) with a momentum of 0.9. The initial learning rate was set to 0.001 and decayed to one-tenth of its current value every 30 epochs. The batch size was set to 4. For the CIFAR-10 dataset, the initial learning rate was set to 0.1 and the batch size was increased to 128, while all other settings remained unchanged. Except for the experiments analyzing the effect of the polarization parameter μ, this parameter was fixed at 0.5 in all experiments. The sparsity factor λ was treated as a dynamically adjusted pruning parameter and tuned according to specific experimental settings. All experiments were implemented using PyTorch version 1.11.0 on an NVIDIA RTX 3090 GPU.

In the experiments, identification accuracy, model computational complexity (measured in terms of floating-point operations, FLOPs), and parameter count were adopted as evaluation metrics for pruning performance, where both FLOPs and parameter counts are measured in millions (M). For comparison, several representative pruning algorithms that have demonstrated strong performance in model pruning were selected, including GAL [35], HRank [46], ARPruning [47], and ASCA [48], to demonstrate the effectiveness of the proposed algorithm.

#### 4.2.1. Analysis of Pruned VGG16 Results on the CIFAR-10 Dataset

The experimental results of pruning the VGG16 network on the CIFAR-10 dataset are summarized in Table 1. To ensure a fair comparison, the identification accuracy of the unpruned VGG16 model is reported as the baseline for all methods, and the FLOPs pruning ratio and parameter pruning ratio achieved by each algorithm are compared. As shown in the table, the proposed algorithm achieves a 70.4% reduction in FLOPs and a 78.3% reduction in parameters, with only a 0.29% decrease in accuracy, demonstrating its effectiveness on the CIFAR-10 dataset.

#### 4.2.2. Analysis of Pruned VGG16 and ResNet18 Results on the IoT Dataset

The pruning results of the VGG16 network on the IoT dataset are presented in Table 2. The results indicate that VGG16 contains significant parameter redundancy, and pruning a portion of the network channels can achieve effective compression with only marginal performance degradation. The proposed AL-PP method with λ=0.01 achieves an identification accuracy of 99.97%, which is comparable to ASCA (99.96%). In contrast, AL-PP (λ=0.05) exhibits a clear advantage in pruning efficiency, further validating the effectiveness of the proposed algorithm for RF fingerprint identification model compression.

Further pruning experiments were conducted on the ResNet18 network, and the results are also reported in Table 2. Compared with VGG16, ResNet18 is a more compact architecture, with significantly fewer FLOPs and parameters. Moreover, ResNet18 consists of multiple residual blocks, each containing two convolutional layers. To avoid dimension mismatch between residual blocks after pruning, consistent with most channel pruning methods, only the first convolutional layer in each residual block was pruned in this study. The sparsity factor λ of the proposed algorithm was set to 0.6 and 0.8. When λ=0.6, the FLOPs and parameter pruning ratios reach 63.70% and 88.89%, respectively, with the FLOPs pruning ratio being noticeably lower than that achieved on VGG16. When λ=0.8, although the model compression capability is significantly improved, the identification accuracy drops to 99.14%, indicating a substantial loss in identification performance. This degradation mainly stems from two factors. First, compared with VGG16, ResNet18 has less inherent parameter redundancy, which limits the number of channels that can be pruned while maintaining accuracy. Second, due to differences in pruning strategies, VGG16 allows channel pruning at every convolutional layer, whereas ResNet18 can only be pruned at the first convolutional layer of each residual block.

**Figure 5:**
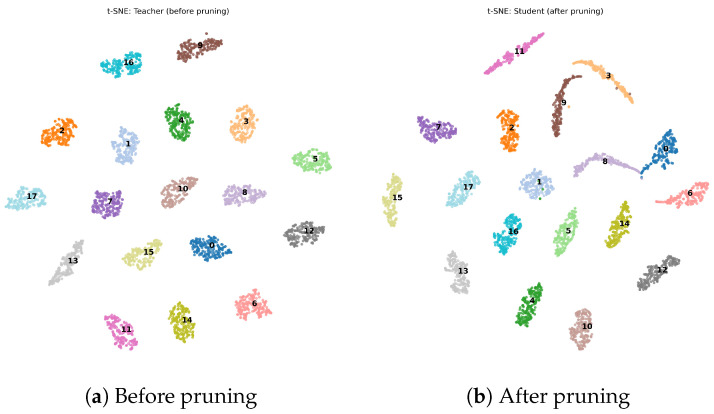
Visualization of data features before and after pruning. The different colors and corresponding numbers represent the unique category labels of the signals used in the experiments.

The features extracted from the final layer of the ResNet18 model were visualized using t-distributed stochastic neighbor embedding (t-SNE), and the results are shown in Figure 5. Figure 5a illustrates the feature distribution before pruning, while Figure 5b shows the feature distribution after pruning.

As can be observed from the figure, the feature clusters before pruning exhibit a more dispersed distribution and varied shapes, indicating that the original model learns rich signal features and achieves strong discriminative capability among signal samples from different devices. After pruning, the feature clusters become more compact in terms of distribution and shape. However, from the perspective of the classification boundaries, the feature clusters corresponding to different devices remain clearly separable, without obvious overlap. This suggests that the pruning operation may lead to the loss of some non-critical feature representations. Overall, the proposed pruning method is able to significantly reduce the model parameter size while effectively preserving the signal identification performance of the model.

#### 4.2.3. Analysis of Pruned VGG and ResNet18 Results on the LoRa Dataset

To verify the effectiveness of the proposed algorithm on different datasets, experiments were conducted on the LoRa dataset, and the results are presented in Table 3. Compared with the IoT dataset, although the LoRa dataset contains fewer classes, each sample has a larger number of sampling points; consequently, both the FLOPs and parameter counts are higher than those of the IoT dataset. When λ=0.01 and λ=0.05, the proposed algorithm achieves significant model compression effects. In terms of accuracy, a slight improvement over the original network is observed, which can be attributed to the more pronounced parameter redundancy of VGG16 on the LoRa dataset. This redundancy makes the original pretrained network prone to overfitting during training, resulting in lower accuracy. By pruning redundant channels, the proposed algorithm alleviates overfitting to some extent and consequently improves the identification accuracy.

The pruning results on ResNet18 are also reported in Table 3. When pruning ResNet18 on the LoRa dataset, a more pronounced degradation in identification accuracy is observed for all methods. This is mainly because the feature distributions among samples in the LoRa dataset are highly similar and less distinguishable, and ResNet18 itself contains limited parameter redundancy. As a result, channel pruning has a greater impact on the model, leading to a faster decline in identification accuracy.

#### 4.2.4. Analysis of Pruned VGG16 Results on the ADS-B Dataset

Finally, to further validate the effectiveness of the proposed pruning algorithm in multi-class identification tasks, VGG16 pruning experiments were conducted on the public ADS-B dataset. The experimental results are summarized in Table 4. Compared with the LoRa and IoT datasets, the ADS-B dataset contains a larger number of classes (28 classes); however, each class includes fewer signal sample points, with only 250 points per sample. Consequently, the FLOPs of the VGG16 network on this dataset are relatively low, amounting to 314.58 M. After pruning the original pretrained model using AL-PP (λ=0.01), the FLOPs and parameter count are reduced by 235.98 M and 11.51 M, respectively, with both the FLOPs pruning ratio and parameter pruning ratio exceeding 75%. When AL-PP (λ=0.05) is applied, both pruning ratios further exceed 80%. These results clearly demonstrate that the proposed algorithm can effectively prune redundant channels in convolutional neural networks, resulting in an RF fingerprint identification model with low memory consumption and high inference efficiency.

### 4.3. Comparison with Lightweight Architectures

To further assess the practical efficiency of the proposed method, we compare AL-PP with several representative lightweight RF fingerprint identification models on the IoT dataset, including MA-TMFN [49], Lightweight CNN [9], and CNMN (ResNet) [50]. The results are reported in Table 5.

Existing lightweight architectures reduce model complexity by redesigning the network structure, whereas the proposed method follows the post-training compression route and improves efficiency by removing redundant channels from a standard backbone. The results show that AL-PP remains competitive when compared with representative lightweight models on the IoT dataset. This suggests that, instead of introducing a new handcrafted lightweight architecture, pruning-based compression can also provide an effective way to balance identification performance and model efficiency.

### 4.4. Ablation Study

To further verify the effectiveness of the pruning method, an ablation study was conducted on ResNet18 using the LoRa dataset, and the results are presented in Table 6. Specifically, the pruning mechanism based on polarization regularization is referred to as Module *A*, the adversarial learning-based performance recovery mechanism is referred to as Module *B*, and the output feature alignment objective is referred to as Module *C*. As shown in Table 6, the adversarial learning-based performance recovery strategy leads to a significant improvement in model accuracy, which clearly demonstrates the effectiveness of this strategy. Moreover, output feature alignment further enhances the identification performance of the model.

### 4.5. Influence of Hyperparameters on Model Pruning

This section analyzes the impact of hyperparameters in the proposed adversarial learning-based polarization pruning algorithm, focusing on the effects of the polarization parameter μ and the sparsity factor λ on the identification accuracy and computational complexity (FLOPs) of the pruned model. The experiments were conducted on the IoT dataset using VGG16 as the pruning backbone. The results are consistent with the role of μ and λ in controlling channel sparsity and feature preservation.

**Figure 6:**
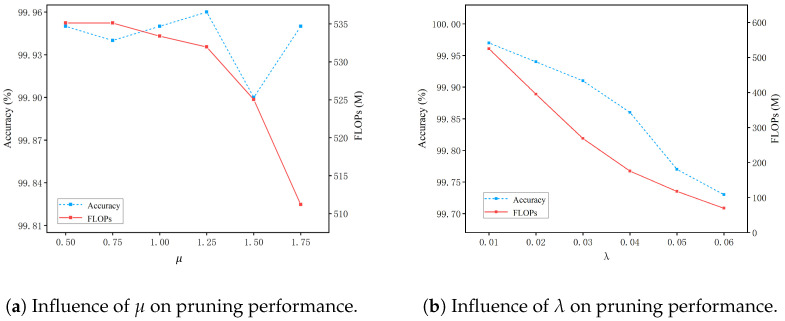
Influence of the polarization parameter μ and the sparsity factor λ on pruning performance of VGG16 on the IoT dataset, measured by identification accuracy and FLOPs.

Figure 6a illustrates the influence of the polarization parameter μ on pruning performance, where the blue curve represents the identification accuracy and the red curve denotes the model FLOPs. As μ increases, the FLOPs gradually decrease. When μ is relatively small, the curve is smooth and the reduction in FLOPs is slow; once μ exceeds 1.25, the FLOPs pruning efficiency improves significantly. This behavior arises because increasing μ strengthens the polarization regularization, further enlarging the disparity among channel importance parameters and pushing more of them toward zero, which allows more redundant channels to be removed. In contrast, the identification accuracy does not monotonically decrease with increasing μ but instead exhibits slight fluctuations. This indicates that, within a certain range, increasing μ can enhance channel separability without causing significant damage to RF fingerprint discriminability, as the accuracy fluctuates only slightly between 99.90% and 99.96%.

The sparsity factor λ is a critical parameter in the proposed pruning algorithm for regulating the network pruning ratio. Figure 6b illustrates the impact of λ on pruning performance. As shown in the figure, with increasing λ, both the identification accuracy and the computational complexity exhibit a monotonically decreasing trend. This behavior arises because λ directly controls the overall strength of the polarization regularization. A larger λ imposes a stronger pruning constraint, leading to more channels being removed in each convolutional layer and a higher degree of model compression, but it may also remove channels that are important for identification. As a result, although stronger regularization improves compression efficiency, it may also cause more noticeable identification accuracy degradation.

To provide a clearer view of the effect of pruning level on model performance, Table 7 summarizes the identification accuracy, FLOPs reduction, and parameter reduction under different values of the sparsity factor λ. As λ increases, the model is compressed more aggressively, and both FLOPs reduction and parameter reduction improve steadily. At the same time, the identification accuracy decreases only slightly, from 99.97% at λ=0.01 to 99.73% at λ=0.06. These results show that the proposed method maintains highly stable identification performance over a wide range of compression levels. Therefore, the added analysis provides a clearer illustration of the trade-off between pruning strength and identification accuracy, and further supports the effectiveness of AL-PP under different pruning settings.

Overall, these results indicate that appropriate selections of μ and λ are necessary to balance structured sparsity and the preservation of discriminative RF fingerprint features, so that redundant channels can be removed without causing significant performance loss.

### 4.6. Analysis of Model Performance Under Noise

**Figure 7:**
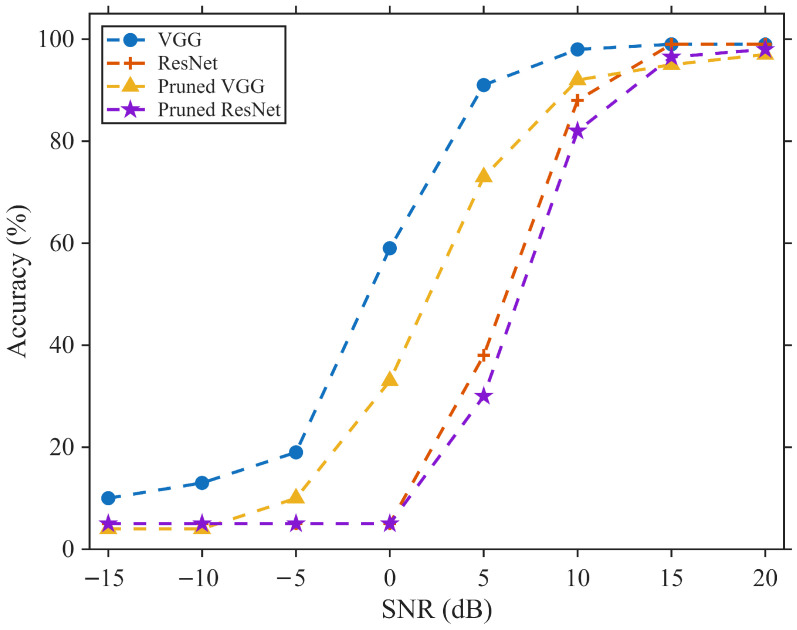
Identification accuracy of networks before and after pruning under different SNR levels.

The pruned RF fingerprint identification models are intended to be deployed on edge embedded devices, where transmitted signals are inevitably affected by noise in practical scenarios. To evaluate the identification performance of the pruned models under noisy conditions, experiments were conducted on the IoT dataset by adding additive white Gaussian noise (AWGN) to the test set to simulate real-world noise interference. The identification accuracies of the networks before and after pruning were compared under different signal-to-noise ratio (SNR) levels. The comparison models include VGG16 and ResNet18, with sparsity factors λ=0.01 and λ=0.6, respectively. Figure 7 illustrates the identification results of each network under SNR conditions ranging from −15 dB to 20 dB.

As shown in the figure, compared with ResNet18, the pruned VGG16 exhibits a larger degradation in identification accuracy under noisy environments. This is mainly because the channel pruning ratio of VGG16 on the IoT dataset is higher than that of ResNet18, which more noticeably affects the network’s ability to extract high-level RF fingerprint features. By comparing the VGG16 network before and after pruning, it can be observed that when the SNR is relatively high (SNR > 10 dB), pruning has a limited impact on noise robustness. As the SNR decreases, the accuracy degradation of the pruned network becomes more pronounced, indicating that some of the pruned channels contribute to capturing subtle signal features affected by noise, an effect that is more evident at lower SNR levels.

Overall, within a certain SNR range, the identification performance of the pruned models does not exhibit a substantial decline, demonstrating that the proposed pruning approach is suitable for practical deployment scenarios.

### 4.7. Performance Evaluation on Embedded Devices

Several types of embedded devices are commonly used in practice, including the NVIDIA Jetson series, Google Coral series, Rockchip series, and the Raspberry Pi series. Among them, the Jetson series is particularly widespread, comprising products such as Jetson Nano, Jetson TX1, and Jetson TX2.

In this section, experiments were conducted on the IoT dataset to compare the identification accuracy and inference time of different models on a central server and on the Jetson TX2 platform. The results are summarized in Table 8, where VGG16-pruned and ResNet18-pruned denote the models obtained using the proposed pruning algorithm. As shown in the table, the identification accuracies of all models on the central server and the Jetson TX2 are largely consistent. Owing to differences in GPU performance, the inference time of each model on the Jetson TX2 is longer than that on the central server. However, through model pruning, the inference time can be significantly reduced, thereby accelerating inference on edge embedded devices.

**Figure 8:**
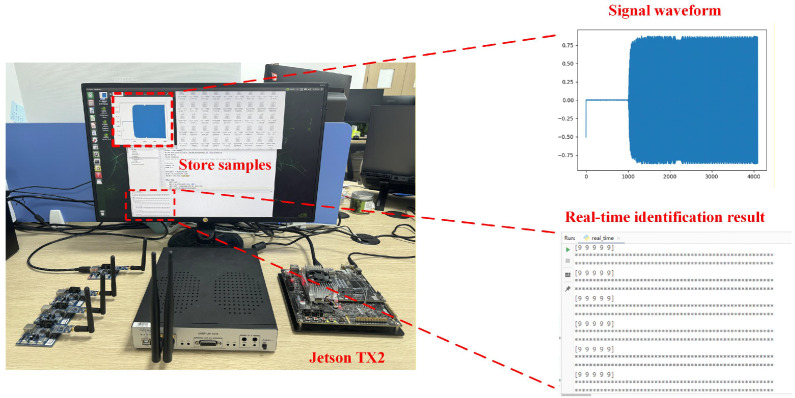
Experimental setup for real-time RFFI evaluation, including signal acquisition, waveform visualization, and identification results on the Jetson TX2 platform.

**Figure 9:**
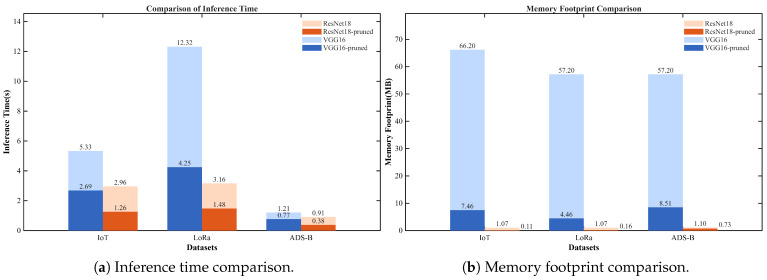
Comparison of inference time and memory footprint on Jetson TX2 before and after pruning.

Furthermore, this section investigates the practical inference performance and memory consumption of the models before and after pruning on the Jetson TX2 platform. The inference time and model parameter memory footprint of VGG16 and ResNet18 were evaluated on the LoRa, ADS-B, and IoT datasets. The experimental setup is shown in Figure 8, and the experimental results are presented in Figure 9, where pruned denotes the pruned models. Compared with the unpruned models, the pruned models exhibit significantly reduced inference time and markedly lower memory usage across all datasets, which facilitates fast inference. In addition, the memory consumption of the pruned models is substantially reduced, facilitating their practical deployment on low-power edge embedded devices.

## 5. Conclusions

Deep learning-based RFFI methods are characterized by excessive model parameters and high computational complexity, which makes them difficult to be directly deployed on edge embedded devices with limited computational and storage resources. Considering the performance trade-off between compression ratio and identification accuracy, this paper proposes a model pruning algorithm based on adversarial learning and polarization regularization to compress deep learning-based RFFI models. The proposed algorithm applies polarization regularization to the model’s soft masks to identify and prune redundant channels. In addition, a performance recovery strategy based on adversarial learning and output feature alignment is introduced to improve the identification accuracy of the pruned model. Experimental results demonstrate that the proposed model pruning algorithm can effectively prune deep learning-based RFFI models with only minor degradation in identification performance, while significantly reducing model parameters and computational cost and improving inference speed. Moreover, the pruned model maintains a high identification accuracy under noisy conditions and exhibits favorable performance when deployed on the embedded Jetson TX2 platform.

## Figures and Tables

**Table 1:** Pruning results of VGG16 on the CIFAR-10 dataset.

Algorithm	Baseline	Accuracy	FLOPs ↓	Parameter ↓
GAL-0.05	93.96%	92.03%	39.6%	77.6%
HRank	93.96%	91.23%	76.5%	92.0%
ARPruning	93.96%	93.18%	72.9%	84.6%
ASCA	93.60%	93.21%	72.8%	71.5%
AL-PP (λ=0.01)	93.12%	92.83%	70.4%	78.3%

**Table 2:** Pruning results of VGG16 and ResNet18 on the IoT dataset.

Model	Algorithm	Accuracy	FLOPs (PR)	Parameters (PR)
VGG16	Base-VGG16	99.99%	4368.79 M (0.00%)	15.00 M (0.00%)
	GAL	99.94%	566.96 M (87.02%)	1.94 M (87.07%)
	HRank	99.45%	587.17 M (86.56%)	1.45 M (90.33%)
	ARPruning	99.93%	334.65 M (92.34%)	1.03 M (93.13%)
	ASCA	99.96%	584.54 M (86.62%)	1.83M (87.80%)
	AL-PP (λ=0.01)	99.97%	525.02 M (87.98%)	1.78 M (88.13%)
	AL-PP (λ=0.05)	99.77%	117.63 M (97.31%)	0.40 M (97.33%)
ResNet18	Base-ResNet18	99.98%	580.90 M (0.00%)	0.27 M (0.00%)
	GAL	99.88%	277.69 M (52.20%)	0.10 M (62.96%)
	HRank	99.31%	230.03 M (60.40%)	0.05 M (81.48%)
	ARPruning	99.75%	168.34 M (71.02%)	0.04 M (85.18%)
	ASCA	99.91%	204.07 M (64.87%)	0.06 M (77.78%)
	AL-PP (λ=0.6)	99.87%	210.87 M (63.70%)	0.03 M (88.89%)
	AL-PP (λ=0.8)	99.14%	142.68 M (75.44%)	0.02 M (92.59%)

**Table 3:** Pruning results of VGG16 and ResNet18 on the LoRa dataset.

Model	Algorithm	Accuracy	FLOPs (PR)	Parameters (PR)
VGG16	Base-VGG16	99.14%	5825.11 M (0.00%)	15.00 M (0.00%)
	GAL	98.42%	948.73 M (83.71%)	2.16 M (85.60%)
	HRank	98.55%	686.18 M (88.22%)	1.48 M (90.13%)
	ARPruning	99.21%	559.21 M (90.40%)	1.27 M (91.53%)
	ASCA	99.30%	743.78 M (87.34%)	1.69 M (88.73%)
	AL-PP (λ=0.01)	99.24%	738.59 M (87.32%)	1.87 M (87.53%)
	AL-PP (λ=0.05)	99.18%	432.10 M (92.58%)	1.07 M (92.87%)
ResNet18	Base-ResNet18	99.97%	790.66 M (0.00%)	0.27 M (0.00%)
	GAL	97.18%	446.96 M (43.47%)	0.16 M (40.74%)
	HRank	97.01%	313.10 M (60.40%)	0.09 M (66.67%)
	ARPruning	98.57%	328.99 M (58.39%)	0.11 M (59.26%)
	ASCA	98.78%	350.51 M (55.67%)	0.15 M (44.44%)
	AL-PP (λ=0.6)	98.66%	353.51 M (55.29%)	0.14 M (48.15%)

**Table 4:** Pruning results of VGG16 on the ADS-B dataset.

Algorithm	Accuracy	FLOPs (PR)	Parameters (PR)
Base	99.14%	314.58 M (0.00%)	15.00 M (0%)
GAL	99.35%	79.31 M (74.79%)	3.54 M (76.40%)
HRank	98.21%	41.05 M (86.95%)	1.56 M (89.60%)
ARPruning	99.26%	36.33 M (88.45%)	1.63 M (89.13%)
ASCA	99.39%	72.19 M (77.05%)	2.97 M (80.20%)
AL-PP (λ=0.01)	99.29%	78.60 M (75.01%)	3.49 M (76.73%)
AL-PP (λ=0.05)	99.28%	52.13 M (83.43%)	2.71 M (81.93%)

**Table 5:** Comparison with Lightweight Architectures on the IoT dataset.

Algorithm	Accuracy	FLOPs	Parameters
MA-TMFN	99.93%	189.62 M	1.81 M
Lightweight CNN	98.17%	658.33 M	1.57 M
CNMN (ResNet)	99.51%	143.62 M	0.64 M
AL-PP (λ=0.01)	99.97%	525.02 M	0.78 M
AL-PP (λ=0.05)	99.77%	117.63 M	0.40 M

**Table 6:** Ablation experiment results.

Method	Accuracy
*A*	97.51%
A+B	98.47%
A+B+C	98.66%

**Table 7:** Identification accuracy and compression ratios for VGG16 under different values of the sparsity factor λ on the IoT dataset.

λ	Accuracy	FLOPs (PR)	Parameters (PR)
Base	99.99%	4368.79 M (0.00%)	15.00 M (0.00%)
0.01	99.97%	525.02 M (87.98%)	1.78 M (88.13%)
0.02	99.94%	378.23 M (91.34%)	1.27 M (91.53%)
0.03	99.92%	261.51 M (94.01%)	0.87 M (94.20%)
0.04	99.86%	170.48 M (96.10%)	0.56 M (96.26%)
0.05	99.77%	117.63 M (97.31%)	0.40 M (97.33%)
0.06	99.73%	68.56 M (98.43%)	0.23 M (98.47%)

**Table 8:** Comparison of model accuracy and inference time of different computing platforms.

Evaluation Index	Model	Central Server	Jetson TX2
Accuracy	VGG16	99.99%	99.99%
	VGG16-pruned	99.91%	99.90%
	ResNet18	99.98%	99.97%
	ResNet18-pruned	99.14%	99.12%
Inference time (ms)	VGG16	4.88	5.33
	VGG16-pruned	2.21	2.69
	ResNet18	2.75	2.96
	ResNet18-pruned	1.01	1.26

## Data Availability

The data presented in this study are available on request from the corresponding author. The data are not publicly available due to the potential leakage of private information.

## Abbreviations

The following abbreviations are used in this manuscript:

IoT	Internet of Things
RFFI	Radio Frequency Fingerprint Identification
BN	Batch Normalization
GAN	Generative Adversarial Network
SGD	Stochastic Gradient Descent
FLOPs	Floating-Point Operations
USRP	Universal Software Radio Peripheral
ADS-B	Automatic Dependent Surveillance–Broadcast
VGG	Visual Geometry Group
t-SNE	t-distributed Stochastic Neighbor Embedding
AWGN	Additive White Gaussian Noise
SNR	Signal-to-Noise Ratio
